# Cucurbit[6]uril‐Derived Nitrogen‐Doped Hierarchical Porous Carbon Confined in Graphene Network for Potassium‐Ion Hybrid Capacitors

**DOI:** 10.1002/advs.202001681

**Published:** 2020-08-26

**Authors:** Daping Qiu, Jingyu Guan, Min Li, Cuihua Kang, Jinying Wei, Feng Wang, Ru Yang

**Affiliations:** ^1^ State Key Laboratory of Chemical Resource Engineering Beijing Key Laboratory of Electrochemical Process and Technology for Materials Beijing University of Chemical Technology Beijing 100029 China; ^2^ Beijing Advanced Innovation Center for Soft Matter Science and Engineering Beijing University of Chemical Technology Beijing 100029 China

**Keywords:** cucurbit[6]uril, energy storage mechanisms, hierarchical porous carbon, nitrogen doping, potassium‐ion hybrid capacitors

## Abstract

Potassium‐ion hybrid capacitors (PIHCs) have attracted tremendous attention because their energy density is comparable to that of lithium‐ion batteries, whose power density and cyclability are similar to those of supercapacitors. Herein, a pomegranate‐like graphene‐confined cucurbit[6]uril‐derived nitrogen‐doped carbon (CBC@G) with ultra‐high nitrogen‐doping level (15.5 at%) and unique supermesopore‐macropores interconnected graphene network is synthesized. The carbonization mechanism of cucurbit[6]uril is verified by an in situ TG‐IR technology. In a K half‐cell configuration, CBC@G anode demonstrates a superior reversible capacity (349.1 mA h g^−1^ at 0.1 C) as well as outstanding rate capability and cyclability. Moreover, systematic in situ/ex situ characterizations, and theory calculations are carried out to reveal the origin of the superior electrochemical performances of CBC@G. Consequently, PIHCs constructed with CBC@G anode and KOH‐activated cucurbit[6]uril‐derived nitrogen‐doped carbon cathode demonstrate ultra‐high energy/power density (172 Wh kg^−1^/22 kW kg^−1^) and extraordinary cyclability (81.5% capacity retention for 5000 cycles at 5 A g^−1^). This work opens up a new application field for cucurbit[6]uril and provides an alternative avenue for the exploitation of high‐performance PIHCs.

## Graphical abstract

An advanced potassium‐ion hybrid capacitor that integrates high energy density, high power density, and outstanding cyclability is constructed with graphene‐confined cucurbit[6]uril‐derived nitrogen‐doped carbon anode and KOH‐activated cucurbit[6]uril‐derived nitrogen‐doped carbon cathode.

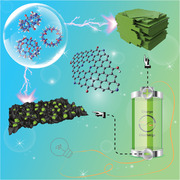

## 1 Introduction

The innovation of energy storage technology is one of the key hooks to alleviate the energy crisis.^[^ ^1^ ^]^ Lithium‐ion batteries (LIBs) with outstanding energy density and supercapacitors (SCs) specializing in power density have been the masters of the energy storage field for decades.^[^ ^2^, ^3^, ^4^ ^]^ However, with the ever‐increasing double demand for energy and power of new large‐scale energy‐consuming devices, neither LIBs nor SCs can shoulder such a heavy task alone.^[^ ^5^, ^6^ ^]^ Alkali metal‐ion (Li^+^, Na^+^, and K^+^) hybrid capacitors, burgeoning energy storage devices that theoretically provide power density and cyclability comparable to SCs without sacrificing energy density, are expected to bridge the gap between LIBs and SCs.^[^ ^5^, ^6^, ^7^ ^]^ Among them, potassium‐ion hybrid capacitors (PIHCs) exhibit broader economic prospects due to the profuse natural abundance of potassium and the similar reduction potential and physical properties of potassium and lithium.^[^ ^8^, ^9^, ^10^, ^11^, ^12^, ^13^, ^14^, ^15^ ^]^ However, PIHCs suffer from severe kinetics failures and cyclability dilemmas since the large ionic size of K^+^ (1.38 Å).^[^ ^10^, ^13^, ^14^, ^15^ ^]^

In the view of PIHCs system, the anode side based on sluggish battery‐type reactions is the short‐board to achieve high‐performance PIHCs.^[^ ^8^, ^9^, ^11^ ^]^ Heteroatom‐doped porous carbon (HDPC) with tunable porosity, abundant electron‐donating heteroatoms, excellent chemical stability, and amorphous carbon framework has been exhibiting promising application prospects in many emerging fields such as energy storage/conversion, catalysis, biomedicine, and gas capture/separation.^[^ ^16^, ^17^, ^18^ ^]^ Acting as PIHCs anode, HDPC not only contributes a considerable reversible specific capacity, but also possesses superior kinetics and cyclability than other types of anode materials.^[^ ^14^, ^15^ ^]^ However, the kinetics of most reported HDPC anodes is still far from matching that of capacitor‐type cathodes. Therefore, developing a novel strategy for the synthesis of HDPC with faster kinetics is highly in demand to further ameliorate the power density and cyclability of PIHCs.

Precursor, the foundation of the structural engineering of HDPC, directly determines the difficulty and feasibility of the synthesis of HDPC.^[^ ^19^, ^20^ ^]^ In principle, the ideal HDPC precursor integrates homogeneous and well‐defined structures, editable frameworks, abundant heteroatoms, and low cost.^[^ ^20^, ^21^, ^22^ ^]^ Cucurbit[*n*]uril (*n* = 5–8) is a kind of N, O‐enriched cage‐like macrocyclic organic compound that can be synthesized through the condensation reaction of glycoluril and formaldehyde (paraformaldehyde).^[^ ^23^, ^24^, ^25^ ^]^ Benefiting from the unique molecular structure, it has attracted extensive interest in many fields, such as supramolecular chemistry, catalysts, and molecular machines.^[^ ^24^, ^25^, ^26^ ^]^ Serving as a HDPC precursor, cucurbit[*n*]uril has outstanding advantages: 1) Abundant N‐ and O‐containing groups, can introduce abundant heteroatoms into carbon framework by self‐doping; 2) Low‐cost raw materials and simple synthetic procedures, are beneficial for large‐scale synthesis of HDPC; 3) Well‐defined molecular structure, reduces the difficulty of tailoring and editing the microstructure of HDPC. Unfortunately, there are almost no reports focusing on cucurbit[*n*]uril‐derived HDPC.

Carbon‐based material confined strategy is an important method widely used in the preparation of conversion anodes and alloy anodes, and has demonstrated profound potential in ameliorating the defects associated with the volume expansion/extraction, agglomeration of nanoparticles, low electrical conductivity, and sluggish ion diffusion.^[^ ^27^, ^28^ ^]^ HDPC anodes suffer from more severe volume expansion/extraction and more sluggish ion diffusion in PIHCs than in lithium‐ and sodium‐ion hybrid capacitors due to the ionic size of K^+^ (1.38 Å) is much larger than that of Li^+^ (0.76 Å) and Na^+^ (1.02 Å).^[^ ^10^, ^29^ ^]^ Drawing on the aforementioned strategy, it is advisable to introduce advanced 2D materials, such as graphene and MXenes, with excellent volume buffering and charge transport properties, to mitigate these dilemmas.^[^ ^30^, ^31^ ^]^

In this work, we have synthesized a unique pomegranate‐like graphene‐confined cucurbit[6]uril‐derived nitrogen‐doped carbon (CBC@G) and successfully applied it to construct PIHCs that integrate high energy density, high power density, and outstanding cyclability. In this pomegranate‐like CBC@G, the cucurbit[6]uril‐derived nitrogen‐doped carbon nanoparticles play the role of electroactive material, and the supermesopore‐macropores interconnected graphene network provides convenient transfer paths for charge and buffers the volume expansion of CBC@G during charge/discharge. Tested as an anode in K half‐cell, CBC@G delivers excellent potassium‐ion storage performances. Furthermore, various in situ, ex situ techniques, and density functional theory (DFT) calculations are carried out to understand the potassium‐ion storage mechanism of CBC@G. As a consequence, PIHCs constructed with CBC@G anode and KOH‐activated cucurbit[6]uril‐derived nitrogen‐doped carbon cathode demonstrate extraordinary energy/power density (172 Wh kg^−1^/22 kW kg^−1^) and cyclability (only 0.0037% capacity decay per cycle at 5 A g^−1^).

## 2 Results and Discussion

**Scheme 1:**
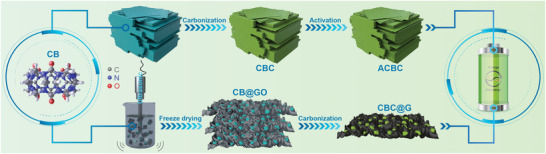
Schematic illustration of the synthesis procedure of CBC@G and CBC.

**Figure 1:**
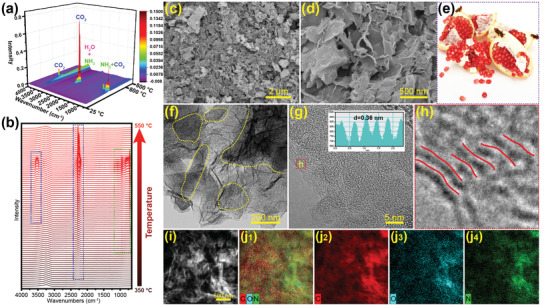
a,b) In situ TG‐IR spectra of cucurbit[6]uril pyrolysis. c,d) SEM images; f–h) TEM images (inset of (g) is the calculated interlayer spacing); i) HAADF; j) EDS elemental mapping of CBC@G. e) Digital photograph of pomegranate.

The synthesis illustration of graphene‐confined cucurbit[6]uril‐derived nitrogen‐doped carbon (CBC@G) and cucurbit[6]uril‐derived nitrogen‐doped carbon (CBC) is displayed in **Scheme** **1**. Nanoplate‐stacked cucurbit[6]uril cubes (Figure S1, Supporting Information, verified by XRD in Figure S2, Supporting Information) and graphene oxide were first dispersed ultrasonically in deionized water to form a homogeneous hydrogel. After freeze‐drying and subsequent pyrolysis, the previously obtained hydrogel was finally converted to pomegranate‐like CBC@G. In situ TG‐IR technology was applied to reveal the carbonization mechanism of cucurbit[6]uril. As shown in Figure S3a, Supporting Information, there are three obvious weightless stages in the TG curve of cucurbit[6]uril at 50–150 °C, 350–550 °C, and 550–780 °C, respectively. Combined with the representative IR spectra of the three stages in Figure S3b, Supporting Information, they are assigned to the volatilization of adsorption and crystal water, carbonization, and removal of functional groups, respectively. Further, it can be observed from **Figure** **1**a that cucurbit[6]uril releases CO_2_ (550–800 cm^−1^, 2150–2420 cm^−1^, 3450–3780 cm^−1^) and NH_3_ (750–1200 cm^−1^, 3150–3500 cm^−1^) during the carbonization stage. These gases are derived from the decomposition of N, O functional groups, and carbon atoms with poor thermal stability in the cucurbit[6]uril skeleton, and the residual skeleton is recombined to form the amorphous CBC. Moreover, as shown in Figure 1b, the carbonization stage of cucurbit[6]uril starts at 350 °C, reaches a maximum rate at 500 °C, and ends at 550 °C. In short, the carbonization stage of cucurbit[6]uril is centered at 350–550 °C, during which the N, O functional groups, and carbon atoms with poor thermal stability are decomposed into CO_2_, NH_3_, and H_2_O to dissipate, and the residual skeleton is recombined to form CBC. The scanning electron microscope (SEM) was conducted to inspect the microstructure of CBC and CBC@G. As shown in Figure S4, Supporting Information, CBC synthesized by direct pyrolysis of cucurbit[6]uril without ultrasonication (synthetic strategy is displayed in Scheme 1) displays a similar nanoplate‐stacked cubes morphology to cucurbit[6]uril, with the size ranging from 2 to 10 µm. In contrast, the SEM images of CBC@G (Figure 1c,d) indicate that the ultrasound‐refined CBC nanoparticles are uniformly anchored on the graphene network. As exhibited in the pomegranate in Figure 1e, CBC@G possesses a pomegranate‐like morphology, where CBC refers to seeds and graphene represents endothelium. In the view of structure, the refinement of particle size is beneficial to the exposure of active sites, and the introduction of graphene network can provide convenient paths for charge transfer.^[^ ^28^, ^32^ ^]^ Further microstructure information was discerned with transmission electron microscope (TEM). The TEM images shown in Figure S5a, Supporting Information, and Figure 1f further confirm the micron‐sized cubic and pomegranate‐like microstructures of CBC and CBC@G, respectively. Notably, the high‐resolution TEM images (Figure S5b, Supporting Information, and Figure 1g,h) reveal that both CBC and CBC@G are typical amorphous carbons with wide carbon interlayer spacing. The widths of the lattice streaks of CBC and CBC@G calculated according to the intensity profile (inset of Figure S5b, Supporting Information, and Figure 1g) is about 0.36 nm.^[^ ^14^ ^]^ Furthermore, the EDS mapping images visualize the element distribution of CBC@G. As exhibited in Figure 1j, the representative C, N, and O elements demonstrate significant segregation in graphene‐confined CBC nanoparticles, which is another expression of the pomegranate‐like structure of CBC@G.

**Figure 2:**
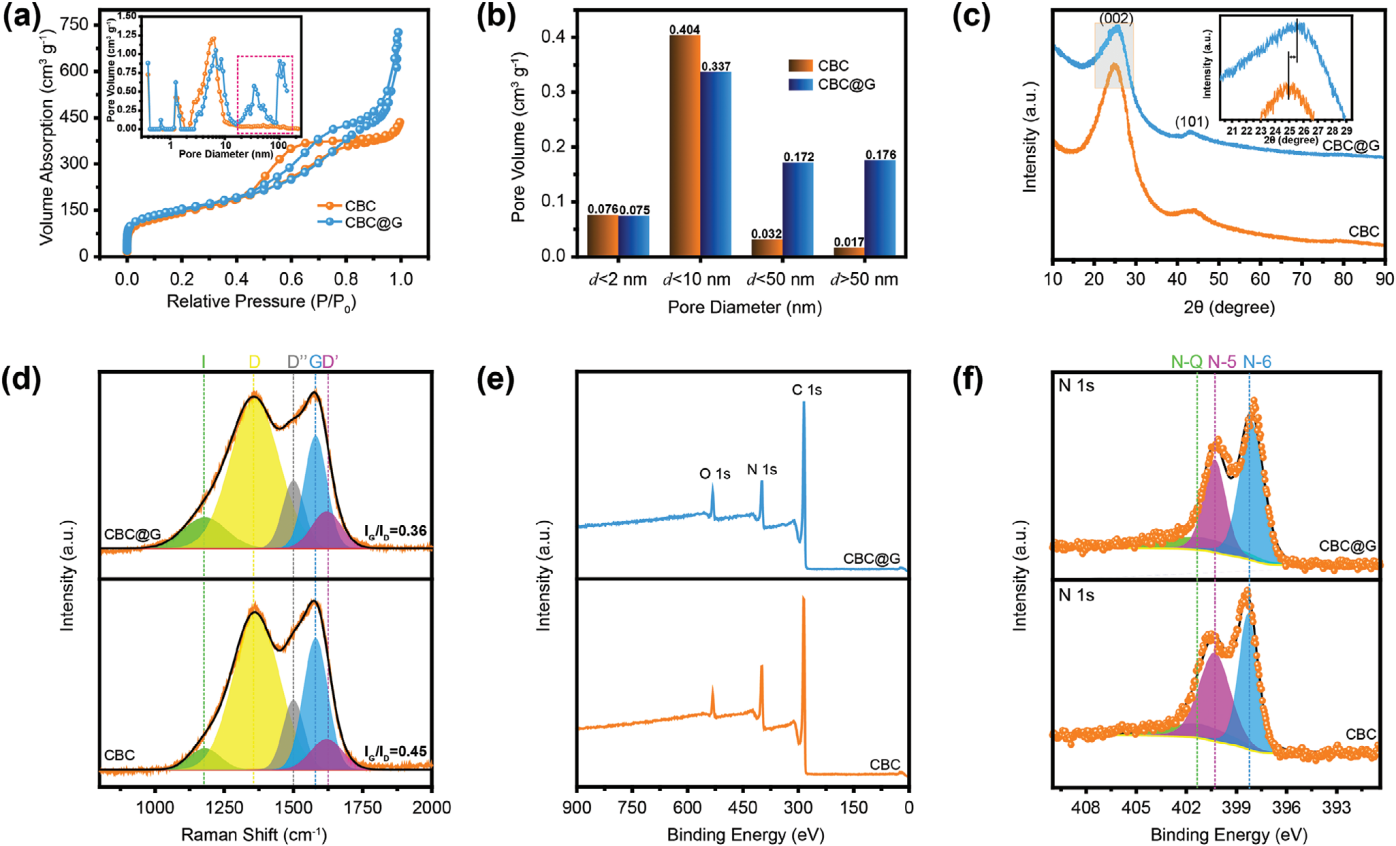
a) N_2_ adsorption–desorption isotherms (inset is the DFT pore size distribution); b) DFT pore size distribution histograms; c) XRD patterns (inset is the partially enlarged patterns); d) Raman spectra; e) XPS survey spectra; f) Deconvolution N 1s spectra of CBC@G and CBC.

N_2_ adsorption–desorption measurements were used to analyze the specific surface areas and porosity of CBC and CBC@G. As shown in **Figure** **2**a, they share similar type IV isotherms with a clear H4 hysteresis loop, confirming the well‐defined mesopores structure.^[^ ^33^, ^34^ ^]^ It is worth mentioning that although they demonstrate similar Brunauer–Emmett–Teller surface area (S_BET_, 509 m^2^ g^−1^ for CBC and 524 m^2^ g^−1^ for CBC@G), their pore size distribution (PSD) is significantly differentiated (inset of Figure 2a, Table S1, Supporting Information). In detail, as displayed in the PSD histogram in Figure 2b, the supermesopore (*d* > 10 nm, 0.172 cm^3^ g^−1^) and macropore (0.176 cm^3^ g^−1^) volume of CBC@G are much higher than those of CBC (0.032 cm^3^ g^−1^ for supermesopore, 0.017 cm^3^ g^−1^ for macropore). Obviously, the introduction of graphene network and ultrasonic refinement are beneficial to the generation of supermesopores and macropores. The crystallographic characteristics of CBC and CBC@G are elucidated by X‐ray diffraction (XRD) and Raman spectroscopy. The XRD patterns (Figure 2c) of CBC and CBC@G exhibit two broad peaks at about 25° and 43°, assigned to the (002) and (101) planes of the graphite crystal, respectively.^[^ ^35^, ^36^ ^]^ Notably, the slightly right‐shifted diffraction angle of the (002) plane (inset of Figure 2c) of CBC@G reveals a slightly narrower interlayer spacing than that of CBC, and the interlayer spacing of CBC@G and CBC calculated by Bragg's Law are 0.350 and 0.355 nm, respectively. The deviation of the carbon interlayer spacing calculated based on the lattice fringes and XRD may be due to the amorphous characteristics of cucurbit[6]uril‐derived carbon. Further, the Raman spectra sensitive to the microstructure of graphite are shown in Figure 2d, where the integrated area ratio (*I*_G_/*I*_D_) of G‐band (1580 cm^−1^, in‐plane bond stretching motion of E_2g_ symmetry) over D‐band (1350 cm^−1^, breathing mode of A_1g_ symmetry) is one of the standard indicators of the graphitization degree of carbonaceous materials.^[^ ^37^ ^]^ The lower *I*_G_/*I*_D_ value of CBC@G (0.36) than CBC (0.45) indicates a lower graphitization degree. The moderate reduction of graphitization degree is beneficial to the exposure of active sites. X‐ray photoelectron spectroscopy (XPS) was carried out to evaluate the elemental configurations of CBC@G and CBC. As depicted in Figure 2e, three peaks of the XPS surveys at about 284.6, 400, and 530 eV are assigned to C, N, and O, respectively.^[^ ^14^, ^38^ ^]^ Surprisingly, the N proportions of CBC@G and CBC are as high as 15.5 at% and 18.5 at%, respectively, higher than most of the reported N‐doped porous carbon.^[^ ^9^, ^14^, ^15^, ^36^ ^]^ The N 1s spectra (Figure 2f) can be deconvolved into pyridinic nitrogen (N‐6), pyrrolic nitrogen (N‐5), and quaternary nitrogen(N‐Q) at 398.4, 400.3, and 401.2 eV, respectively.^[^ ^38^, ^39^ ^]^ The deconvolution spectra of C 1s and O 1s are shown in Figure S6, Supporting Information, confirming the existence of abundant N, O groups in CBC@G and CBC (see details in the Supporting Information). Electroactive groups such as N‐6, N‐5, C = O can contribute additional pseudocapacitance to carbonaceous materials, and other groups such as N‐Q, C‐O are beneficial for intrinsic conductivity.^[^ ^40^, ^41^ ^]^ Further, as shown in the histogram (Figure S7, Supporting Information), the atomic and relative contents of N‐6 of CBC@G are higher than those of CBC. As reported, the pseudocapacitance contributed by N‐6 is more reversible than that contributed by N‐5.^[^ ^41^, ^42^ ^]^ In short, the pomegranate‐like CBC@G demonstrates higher supermesopore and macropore volume, more disordered carbon framework, slightly narrow interlayer spacing, and higher N‐6 content than CBC synthesized by direct pyrolysis.

**Figure 3:**
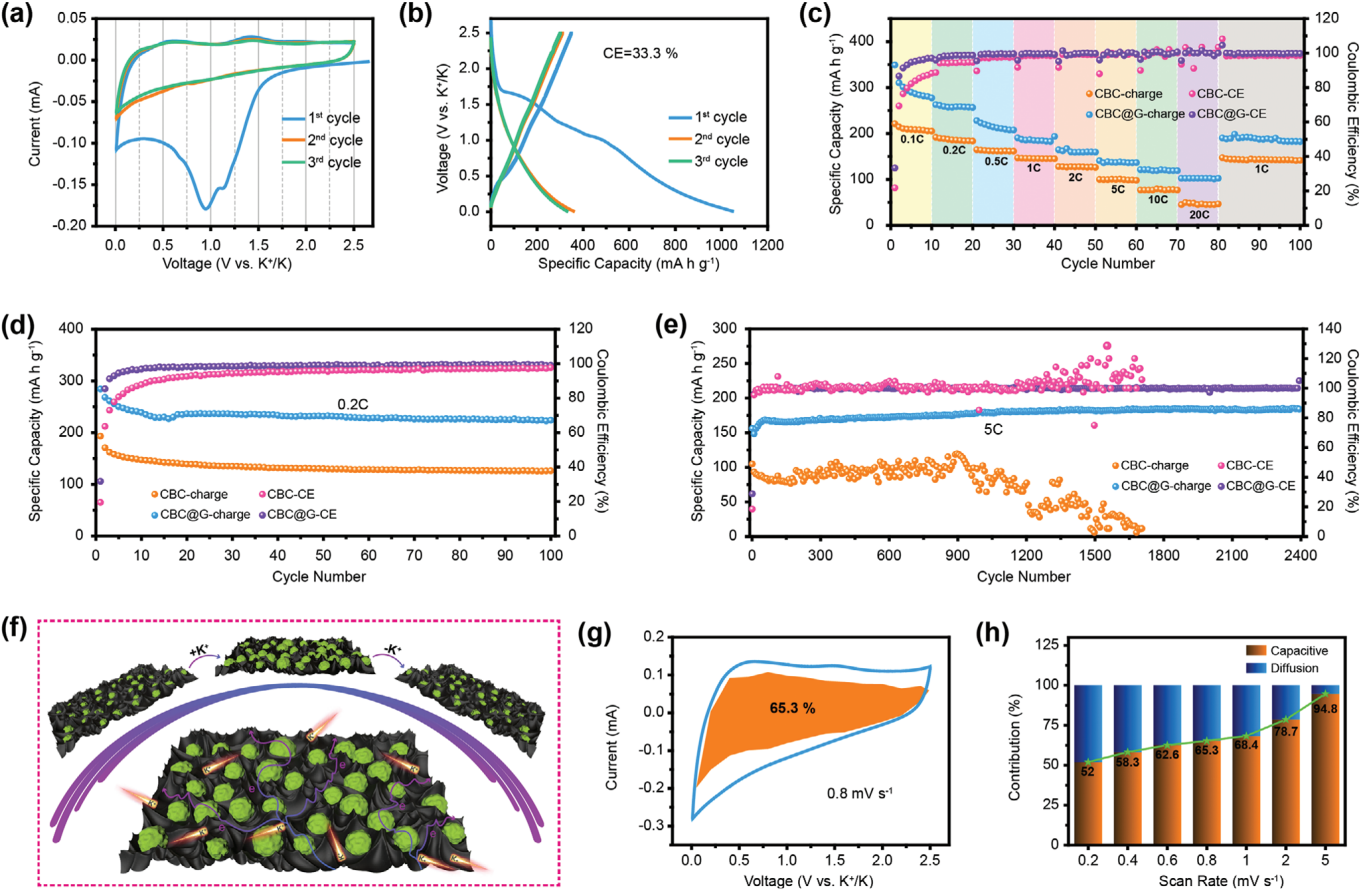
Electrochemical properties of CBC@G and CBC. a) CV curves of the first three cycles of CBC@G at 0.1 mV s^−1^. b) GCD curves of the first three cycles of CBC@G at 0.1 C (1 C = 280 mA g^−1^). c) Rate capability, d) cycling stability at 0.2 C, e) ultralong cycling stability at 5 C of CBC@G and CBC. f) Schematic illustration of the potassium‐ion storage mechanism of CBC@G. g) Capacitive behavior (orange region) and battery‐behavior (white region) contributions at 0.8 mV s^−1^, h) normalized contribution ratio of capacitive behavior and battery‐behavior capacities at different scan rates of CBC@G.

The potassium‐ion storage properties of CBC@G and CBC were evaluated by means of cyclic voltammetry (CV) and galvanostatic charge–discharge (GCD) techniques. **Figure** **3**a exhibits the initial three cyclic CV curves for CBC@G anode at 0.1 mV s^−1^. The irreversible cathodic peak of the first cycle around 0.95 V may originate from the irreversible reactions and the generation of solid electrolyte interface layer (SEI).^[^ ^43^ ^]^ Further, the CV curves of CBC@G almost coincides in subsequent cycles, unveiling its excellent electrochemical reversibility. Notably, the CV curves of CBC@G after the first cycle all present a distorted rectangular shape, which may indicate that the capacitive behavior dominates the energy storage mechanism of CBC@G.^[^ ^44^ ^]^ The GCD curves of CBC@G for the initial three cycles at 0.1 C (1 C = 280 mA g^−1^) are shown in Figure 3b. Consistent with the CV curve of the first cycle, the plateau of the first cyclic GCD curve of CBC@G at 1.6–0.75 V corresponds to the irreversible reactions and the generation of SEI. This significant irreversible plateau resulted in a slightly frustrated initial coulombic efficiency (CE) of CBC@G (33.3%). But it is commendable that the initial CE of pomegranate‐like CBC@G has ameliorated significantly compared to CBC (21.8%, shown in Figure S8, Supporting Information). Detailed rate capability data for CBC@G and CBC are depicted in Figure 3c. CBC@G delivers an initial reversible specific capacity of 349.1 mA h g^−1^ with an initial CE of 33.3% at 0.1 C, maintains 102.5 mA h g^−1^ at an ultra‐high current density of 20 C, the rate capability are superior to many reported carbon‐based potassium‐ion battery anodes (Figure S9a, Supporting Information). In addition, CBC@G can still contribute a high reversible specific capacity of 190 mA h g^−1^ when the current density returns to 1 C. As a comparison, CBC can only achieve a suppressed reversible specific capacity of 221.2 mA h g^−1^ with an initial CE of 21.8% at 0.1 C, and the corresponding value is as low as 45.1 mA h g^−1^ at 20 C. Cycling stability is one of the key parameters of PIHCs anode. As displayed in Figure 3d,e, CBC@G can hold 88% and 100% of the reversible specific capacity with nearly 100% CE after 100 and 2400 cycles at 0.2 and 5 C, respectively. The abnormal recovery of the specific capacity of CBC@G at the beginning of 5 C may be due to the incomplete wetting of the electrode in electrolyte during the initial tens of cycles.^[^ ^43^ ^]^For CBC, despite the capacity retention at low current density (0.2 C) is considerable, its cycling stability at high current density (5C) is unsatisfactory (the reversible capacity is close to 0 mA h g^−1^ after about 1600 cycles). Furthermore, the SEM images and XRD pattern of CBC@G anode after 2400 cycles at 5 C are shown in Figure S10, Supporting Information, in which the SEM images without obvious cracks confirms the robust mechanical stability, and the still detectable (002) plane (graphite crystal) in the XRD pattern reveals outstanding structural stability. In addition, as shown in Figure S11, Supporting Information, CBC@G displays excellent wettability to electrolyte (the contact angle after 10 s is close to 0°), which is probably ascribed to abundant electrolyte‐friendly groups of CBC@G.^[^ ^45^ ^]^ In short, the extraordinary potassium‐ion storage properties of CBC@G can be attributed to the following three aspects (Figure 3f): 1) The abundant exposed active sites and prominent N‐6 content significantly improve the reversible specific capacity; 2) The supermesopores‐macropores interconnected graphene network provides convenient paths for charge transfer, thus ameliorating the rate capability; 3) The graphene network with robust mechanical strength buffers the volume expansion of CBC@G during charge/discharge, resulting in superb cycling stability.

**Figure 4:**
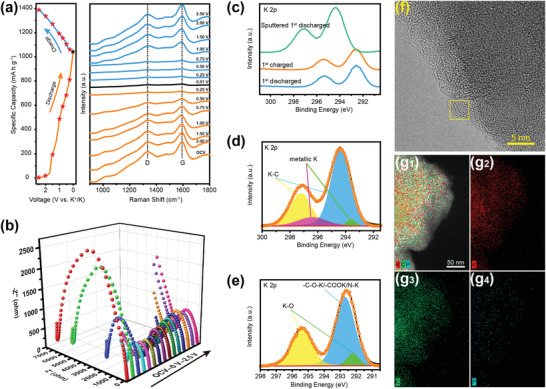
Potassium‐ion storage mechanism of CBC@G. a) In situ Raman spectra. b) In situ EIS of CBC@G in the first discharge/charge process. c) K 2p high‐resolution XPS of CBC@G at different discharge/charge states. d) Deconvolution K 2p spectrum of fully discharged CBC@G after Ar ion sputtering. e) Deconvolution K 2p spectrum of fully charged CBC@G. f) TEM image of fully discharged CBC@G. g) EDS elemental mapping of fully charged CBC@G.

Kinetic analysis, in situ/ex situ techniques, and DFT calculations were carried out to understand the potassium‐ion storage mechanism of CBC@G. The Dunn theory was employed to quantify the proportion of capacitive‐behavior and battery‐behavior in CBC@G‐based electrodes. In such context, the proportion of the above two behaviors can be evaluated according to the following equation:^[^ ^46^ ^]^

(1) $$\begin{array}{c} i\left(V\right)=k_{1}\nu +k_{2}\nu^{1/2} \end{array}$$

where *i*, *v*, k_1_ *ν*, and k_2_ *ν* ^1/2^ represent the current response at the selected potential (*V*), the scan rate of the CV curve (Figure S9b, Supporting Information), the contribution of capacitive‐behavior, and the contribution of battery‐behavior, respectively. As shown in Figure 3g, the capacitive‐behavior content (orange region) of CBC@G at 0.8 mV s^−1^ is about 65.3%. Further, as can be observed in Figure 3h, the capacity contributed by capacitive‐behavior is steadily increased from 52% to 94.8% as the scan rate is increased from 0.2 to 5 mV s^−1^. As a comparison, the corresponding values of CBC (Figure S10d, Supporting Information) at each scan rate are significantly lower than those of CBC@G. Such significant capacitive‐behavior dominated energy storage behavior of CBC@G may be attributed to the ultrahigh proportion of electroactive N‐containing groups.^[^ ^41^ ^]^ Galvanostatic intermittent titration (GITT) was conducted to calculate the diffusion coefficient of K^+^ (*D*_k_) in CBC@G. According to the calculation principle in Figure S12a, Supporting Information, CBC@G demonstrates potassiation/depotassiation *D*_k_ values of up to 8 × 10^−11^ cm^2^ s^−1^ (Figure S12b, Supporting Information), which reveals the rapid K^+^ diffusion kinetics of CBC@G.^[^ ^47^ ^]^ In situ Raman spectroscopy and in situ electrochemical impedance spectroscopy (EIS) were applied to investigate the charge/discharge process of CBC@G. As displayed in **Figure** **4**a, the D‐band and G‐band of CBC@G gradually weaken with the deepening of the discharge state, and almost disappear when fully discharged. The continuous intercalation of K^+^ promotes the conversion of the original CBC@G to the K‐C compounds, resulting in the atrophy of the D‐band and G‐band. During the charge process, the nearly disappeared D‐band and G‐band are rejuvenated, and restore to the original states at 2.5 V.^[^ ^43^ ^]^ Obviously, the previously generated K‐C compounds are reconverted to CBC@G during the charge process. The in situ EIS for the first discharge/charge cycle of CBC@G is depicted in Figure 4b, where the diameter of the semicircle in the high frequency region deforms significantly at different charge/discharge stages. In general, the diameter of this semicircle refer to the diffusion resistance of K^+^ (DSR).^[^ ^48^, ^49^ ^]^ It can be observed that the DSR of CBC@G gradually decreases with the deepening of the discharge state, while the corresponding value slowly increases with the deepening of the charge state. Obviously, the intercalation of K^+^ is conducive to the ionic conductivity of CBC@G. Notably, CBC@G demonstrates a large DSR at initial stage of discharge, which is due to the absence of fast‐ionic conductor interface for the pristine electrode.^[^ ^49^ ^]^ The reversible transition of the Raman spectrum and EIS during a complete discharge/charge cycle reveals the highly reversible potassiation/depotassiation process of CBC@G. Ex situ XPS and ex situ high‐resolution TEM were used to detect the configuration of K^+^ inside the carbon framework at different charge/discharge states. As shown in Figure 4c, the fully discharged (blue spectrum) and fully charged (orange spectrum) electrodes without Ar ion sputtering exhibit a similar K 2p spectrum, while the K 2p spectrum of the fully discharged electrode is significantly distorted after Ar ion sputtering (green spectrum). Considering the detectable depth of XPS, the K 2p spectrum detected before Ar ion sputtering belongs to the SEI at the electrode interface, and the K 2p spectrum after Ar ion sputtering treatment corresponds to the product of K^+^ intercalation.^[^ ^50^ ^]^ The K 2p spectrum of the fully discharged electrode after Ar ion sputtering can be deconvoluted into the dominant K‐C and secondary metallic K (Figure 4d), where K‐C is assigned to the graphite intercalation compound (KC*_x_*) and metallic K maybe be attributed to the adsorbed K^+^.^[^ ^51^, ^52^ ^]^ The coexistence of K‐C and metallic K in the K 2p spectrum further confirmed the hybrid energy storage mechanism of CBC@G. Reflected in the high‐resolution TEM image (Figure 4f), the fully discharged CBC@G demonstrates a significantly expanded carbon interlayer spacing (Figure S13, Supporting Information). Notably, the carbon interlayer spacing of the fully charged CBC@G (Figure S14, Supporting Information) can be restored to 0.36 nm, which is basically consistent with the pristine sample, further confirming the highly reversible potassiation/depotassiation process of CBC@G. Furthermore, the K 2p spectrum of the fully charged electrode was selected to evaluate the components of SEI. As presented in Figure 4e, the deconvolution K 2p spectrum of the fully charged electrode is assigned to K‐O (292.2 eV, 295.4 eV) and —C—O—K/COOK/N—K (292.7 eV).^[^ ^52^, ^53^ ^]^ Combining the analysis results of the corresponding deconvolution spectra of C 1s and O 1s (Figure S15, Supporting Information), it can be seen that K_2_CO_3_, K_2_O, and K‐containing organic compounds are the dominant components of the SEI of CBC@G. In addition, as depicted in Figure 4g, the uniformly distributed K in EDS mapping confirms the presence of K‐compounds based SEI. DFT calculations were performed to get insight into the N‐doping effect on the performance of CBC@G. The K‐adsorption abilities of the graphene layers based on different N‐doping modes are shown in Figure S19a–e, Supporting Information. First, the K‐adsorption energy (Δ*E*) of defect graphene layer without N‐doping (−2.700 eV, Figure S19a, Supporting Information) is significantly higher than that of N‐5 or N‐6 doped graphene layer, confirming the positive effect of N‐doping on the performance of carbon materials. Further, among graphene layers with different N‐doping modes, N‐5 and N‐6 co‐doped graphene layers (Figure S19c,d, Supporting Information) exhibit lower Δ*E* than graphene layers doped with N‐5 or N‐6 alone(Figure S19b,e, Supporting Information). Notably, as shown in Figure S19f, Supporting Information, the N‐6 dominant N‐5 and N‐6 co‐doped graphene layer possesses the lowest Δ*E* (2N6, −3.407 eV). Obviously, the N‐6 dominant N atom configuration of CBC@G (N‐6/N‐5 = 7.94/4.96) is favorable for its potassium‐ion storage properties.

**Figure 5:**
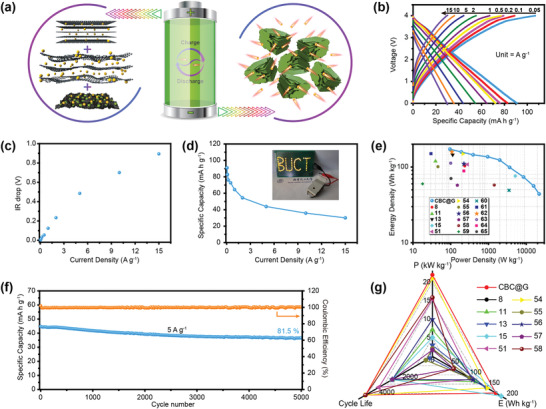
Electrochemical properties of CBC@G//ACBC PIHCs. a) Schematic illustration of CBC@G//ACBC PIHCs. b) GCD curves. c) IR drop. d) Rate capability at different current densities (inset is the digital photograph of LEDs powered by CBC@G//ACBC PIHCs). e) Ragone plots. f) Cycling stability at 5 A g^−1^. g) Comparison of electrochemical performance indices with other reported PIHCs.

To evaluate the application prospect of CBC@G in PIHCs, a PIHCs device (CBC@G//ACBC) with CBC@G as the anode and KOH‐activated CBC (ACBC, see discussion details in the Supporting Information) as the cathode has been constructed (**Figure** **5**a). As revealed in Figure S20, Supporting Information, the CV curve of the CBC@G//ACBC exhibits a quasi‐rectangular shape without obvious polarization, indicating that it can be operated steadily at an operating voltage of 0.01–4 V. The typical GCD curves of the CBC@G//ACBC at different current densities are displayed in Figure 5b and the corresponding reversible specific capacities are summarized in Figure 5d. The CBC@G//ACBC can deliver an ultra‐high reversible specific capacity of 91 mA h g^−1^ with a quasi‐linear GCD curve at 0.05 A g^−1^, and can still contribute 30.1 mA h g^−1^ with an ultra‐short discharge time of 7.2 s even at 15 A g^−1^. Notably, the IR drop of the CBC@G//ACBC is only 0.89 V even at an ultra‐high current density of 15 A g^−1^ (Figure 5c), which reveals the excellent kinetics of the CBC@G//ACBC system. The Ragone plot is depicted to reflect the energy/power density index of the CBC@G//ACBC. As presented in Figure 5e, the CBC@G//ACBC PIHCs can achieve an ultra‐high energy density of 172 Wh kg^−1^ at 172 W kg^−1^, and supply a considerable energy density of 44 Wh kg^−1^ even at an ultra‐high power density of 22 kW kg^−1^. Both energy density and power density are comparable to or even exceed most of the reported lithium‐/sodium‐/PIHCs.^[^ ^8^, ^11^, ^13^, ^15^, ^51^, ^54^, ^55^, ^56^, ^57^, ^58^, ^59^, ^60^, ^61^, ^62^, ^63^, ^64^, ^65^ ^]^ In terms of cycling stability, the CBC@G//ACBC device delivers an extraordinary cycling stability with a capacity retention of 81.5% over 5000 cycles at 5 A g^−1^, only 0.0037% capacity decay per cycle (Figure 5f and Figure S21, Supporting Information). In addition, a CBC@G//ACBC fully charged at 5 A g^−1^ can easily light up a “BUCT” panel welded by yellow light‐emitting diodes (inset of Figure 5d). The disassembled parts of CBC@G//ACBC after 5000 cycles are shown in Figure S22, Supporting Information, where the separator still maintains the original hue and the active materials are almost not peeled off, further confirming its excellent cycling stability. Moreover, as shown in the radar map (Figure 5g), the comprehensive indexes of energy density, power density, and cycling stability of CBC@G//ACBC PIHCs are superior to most of the reported PIHCs.^[^ ^8^, ^11^, ^13^, ^15^, ^51^, ^54^, ^55^, ^56^, ^57^, ^58^ ^]^

## 3 Conclusion

In summary, a unique pomegranate‐like graphene‐confined cucurbit[6]uril‐derived nitrogen‐doped carbon (CBC@G) has been fabricated through an ultrasound‐assisted strategy. Benefiting from the unique structural features of the refined carbon nanoparticles, expanded carbon interlayer spacing, amorphous carbon framework, ultra‐high N content (15.5%), and supermesopores‐macropores interconnected graphene network, CBC@G anode demonstrates excellent potassium‐ion storage properties. Furthermore, systematic in situ/ex situ characterizations, and DFT calculations have been carried out to unveil the origin of the superior electrochemical properties of CBC@G. As a consequence, the high‐performance PIHCs assembled with CBC@G anode and ACBC cathode deliver an ultrahigh energy density of 172 Wh kg^−1^ at 172 W kg^−1^, and remain as high as 44 Wh kg^−1^ even at an ultra‐high power density of 22 kW kg^−1^. In addition, the as‐assembled PIHCs exhibit excellent cycle life with 81.5% capacity retention after 5000 cycles at 5 A g^−1^. We believe that this work opens up a new application field for cucurbit[6]uril and provides an alternative avenue for the further development of high‐performance PIHCs.

## 4 Experimental Section

##### Materials

All analytical grade chemicals were purchased from Shanghai Aladdin Biochemical Technology Co., Ltd., and used without further purification.

##### Materials Preparation

Cucurbit[6]uril was synthesized according to the literature procedures with slight modifications.^[^ ^25^ ^]^ 2 g of cucurbit[6]uril was dispersed in 100 mL of GO aqueous solution (2 mg mL^−1^) under stirring, followed by ultrasonic treatment for 6 h for complete dispersion of the components. The mixture was then fully frozen by refrigerator and freeze‐dried for 48 h to obtain CB@GO. CB@GO was placed into a tube furnace for carbonation at 700 °C (heating rate = 5 °C min^−1^) for 5 h under Ar atmosphere. The obtained CBC@G was washed with deionized water for several times, and finally dried in an oven at 70 °C. CBC was obtained by directly carbonizing cucurbit[6]uril under the same conditions. 1 g of CBC and 4 g of KOH were added to deionized water successively, stirring continuously until homogeneous slurry was formed. After drying in an oven, the residue was activated at 800 °C for 2 h in an Ar atmosphere. The activated residues (ACBC) were first washed with 1 m HCl to remove the soluble potassium salt, then thoroughly washed with deionized water until filtrate became neutrality, and finally dried in an oven overnight.

##### General Characterization

X‐ray diffractometer (XRD, Rigaku RINT 2200) and Raman spectrometer (LabRAM HR800) were employed to character structural information, and in situ Raman spectra were tested at the excitation wavelength of 532 nm. The morphology information was characterized on SEM (S‐4800, Hitachi) and TEM (FEI TECNAI G2 F20). XPS was collected on an ESCALAB 250 XPS system (Thermo Fisher). Specific surface area was tested by N_2_ adsorption–desorption isotherms (Micromeritics, ASAP 2020). The porosity was obtained by the DFT method. The CBC@G electrodes at different charge/discharge states were carefully cleaned by DEC in the Ar‐filled glovebox, then put into sealed glass bottles to avoid exposure to air.

##### Electrochemical Measurements

The active material, Super P, and sodium alginate were uniformly ground in H_2_O at a mass ratio of 8: 1: 1, coated onto collector (Cu foil for anode, Al foil for cathode) and dried at 120 °C for 12 h in a vacuum oven. CR2032 coin‐type cells were assembled in an Ar‐filled glovebox (H_2_O, O_2_ < 0.1 ppm). K metal was used as the counter electrode for half‐cells, and ACBC was selected as PIHCs cathode (the active material mass ratio of cathode and anode was fixed as 1:2). Glass fibers (Whatman, GF/D) and 0.8 m KPF_6_ in EC: DEC (1: 1 in volume) were employed as separator and electrolyte, respectively. Before the assembly of PIHCs, CBC@G anode was prepotassiated 3 times in a half‐cell at 0.2 C. GCD tests were finished using a CT2001A cell test system (Wuhan LAND). The CV and in situ EIS studies were carried out on an electrochemical workstation (CHI 760E). GITT was performed under 0.1 C pulses for 30 min, and then relaxed for 1 h. The calculations of energy (*E*, Wh kg^−1^) and power density (*P*, W kg^−1^) were performed using the following equations:

(2) $$\begin{array}{c} E\;=\int_{t_{1}}^{t_{2}}UI/mdt \end{array}$$

(3) $$\begin{array}{c} P\;=\frac{E}{t}\;\times \;3600 \end{array}$$

where *I* (A), *U* (V), *m* (g), *t*_1_, and *t*_2_ (s) are assigned to the discharge current, operating voltage, total mass of active materials, start and end times of the discharge, respectively, and *t* is *t*_1_–*t*_2_ (s).

##### Calculation Details

The calculations implemented by the Vienna Ab initio Simulation Package code based on DFT, with the projector‐augmented wave potentials and generalized gradient approximation of Perdew–Burke–Ernzerhof. The models were constructed by 6 × 6 × 1 graphite‐like carbon supercell with defects, 3N‐5, 1N‐6/2N‐5, 2N‐6/1N‐5, N‐6, as shown in Figure S19, Supporting Information. Then a 450 eV energy cutoff and 1 × 2 × 1 Monkhorst‐Pack k‐points were set to optimize the structures. The atomic positions were relaxed until the forces on the atoms were smaller than 0.01 eVÅ^−1^.

## Conflict of Interest

The authors declare no conflict of interest.

## Supporting information

Supporting Information
