# Supplementary material for: Cucurbit[6]uril‐Derived Nitrogen‐Doped Hierarchical Porous Carbon Confined in Graphene Network for Potassium‐Ion Hybrid Capacitors
Source: Adv Sci (Weinh). 2020 Aug 26;7(20):2001681. doi: 10.1002/advs.202001681 (PMC7578902; doi:10.1002/advs.202001681)
Supplement: Supplementary file 1 — Supporting Information [file ADVS-7-2001681-s001.pdf]

© 2020 Wiley-VCH GmbH

ADVANCED SCIENCE
Open Access

Supporting Information

for *Adv. Sci.*, DOI: 10.1002/advs.202001681

Cucurbit[6]uril-Derived Nitrogen-Doped Hierarchical Porous Carbon Confined in Graphene Network for Potassium-Ion Hybrid Capacitors

Daping Qiu, Jingyu Guan, Min Li, Cuihua Kang, Jinying Wei, Feng Wang, and Ru Yang**

Supporting Information

Cucurbit[6]uril-Derived Nitrogen-Doped Hierarchical Porous Carbon Confined in Graphene Network for Potassium-Ion Hybrid Capacitors

Daping Qiu, Jingyu Guan, Min Li, Cuihua Kang, Jinying Wei, Feng Wang, and Ru Yang**

D. Qiu, J. Guan, Dr. M. Li, C. Kang, J. Wei, Prof. F. Wang, Prof. R. Yang
State Key Laboratory of Chemical Resource Engineering, Beijing Key Laboratory of Electrochemical Process and Technology for Materials
Beijing University of Chemical Technology
Beijing 100029, China
E-mail: ruyang@mail.buct.edu.cn (R. Yang); wangf@mail.buct.edu.cn (F. Wang)

Prof. F. Wang
Beijing Advanced Innovation Center for Soft Matter Science and Engineering
Beijing University of Chemical Technology
Beijing 100029, China.

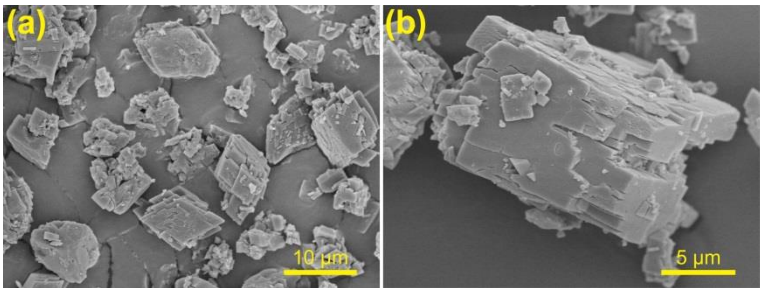

Figure. S1 SEM images of cucurbit[6]uril.

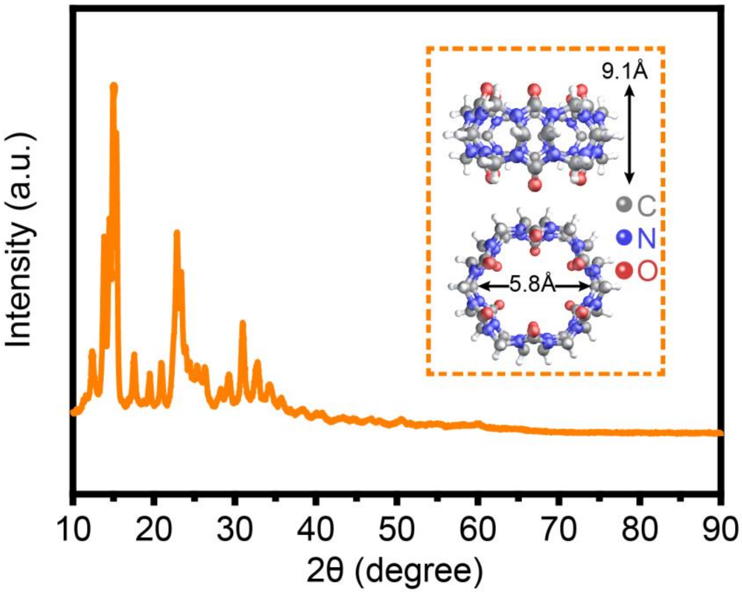

Figure. S2 XRD pattern of cucurbit[6]uril.

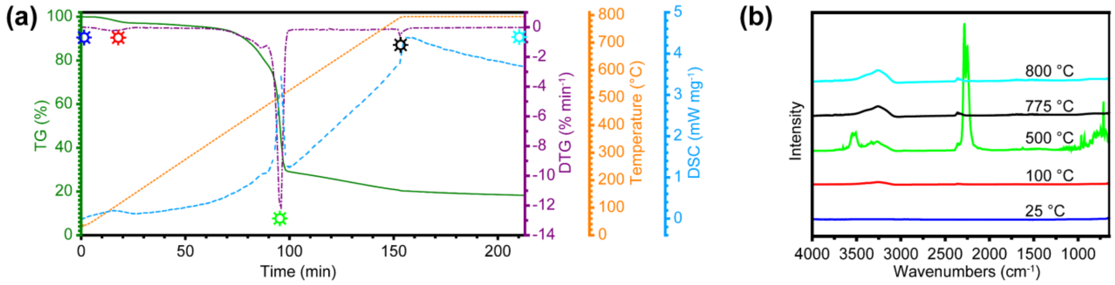

Figure. S3 (a) TG-DTG-DSC curve of cucurbit[6]uril, (b) In-situ TG-IR spectra of cucurbit[6]uril pyrolysis.

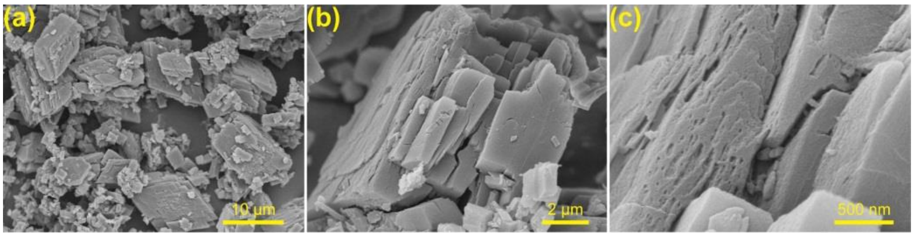

Figure. S4 SEM images of CBC.

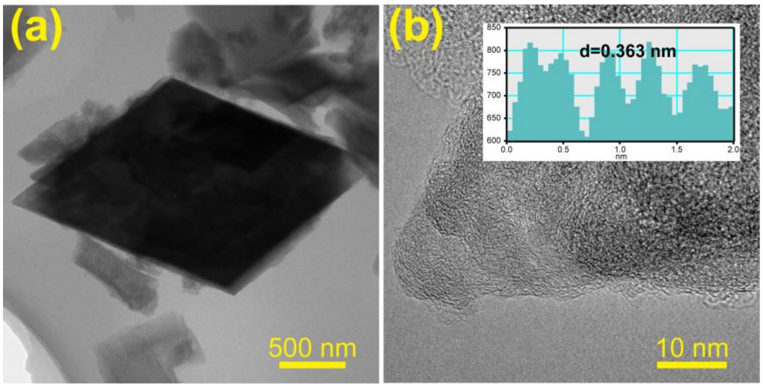

Figure. S5 TEM images of CBC.

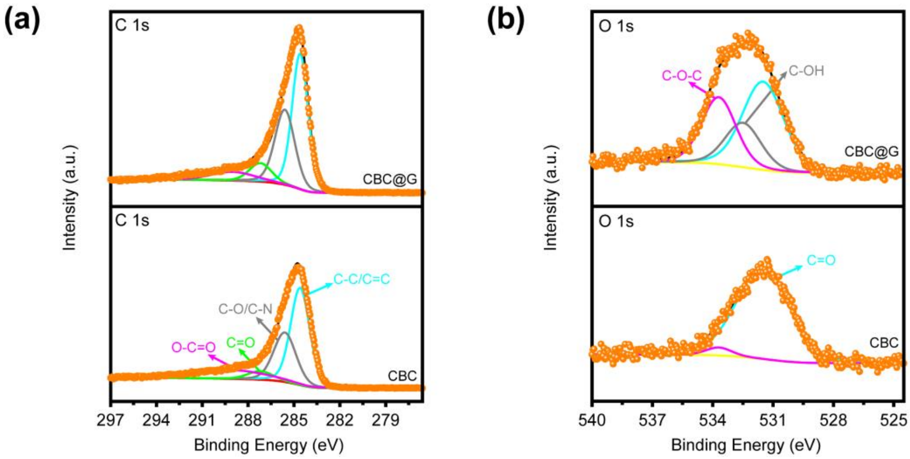

Figure. S6 (a) Deconvolution C 1s spectra, (b) deconvolution N 1s spectra of CBC@G and CBC.

The deconvolution C 1s spectra of CBC@G and CBC displays four types of binding around 284.6, 285.6, 287.2, and 289 eV, representing C-C/C=C, C−O/C−N, C=O, and O-C=O, respectively.[6] As for the spectra of O 1s, the directly pyrolyzed CBC only contains C=O (531.5 eV) and C-O-C (533.7 eV), while the pomegranate-like CBC@G additionally contains C-OH (532.5 eV).[7] Obviously, the introduction of graphene network and ultrasonic refinement have introduced more abundant O-containing groups.

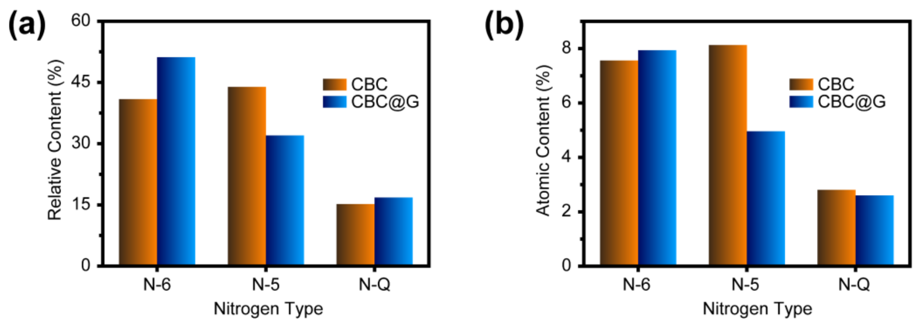

Figure. S7 (a) Relative content, (b) atomic content of N in CBC@G and CBC.

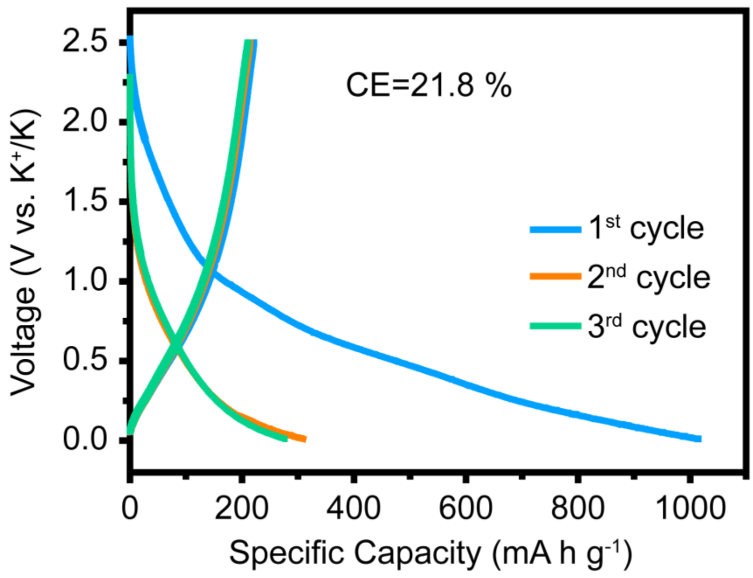

Figure. S8 GCD curves of the first three cycles of CBC at 0.1C.

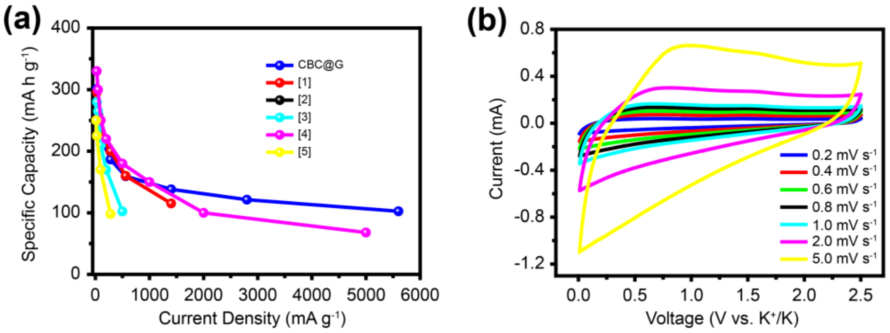

Figure. S9 (a) Comparison of CBC@G rate capability with other carbon-based anodes, (b) CV curves of CBC@G at different scan rates.

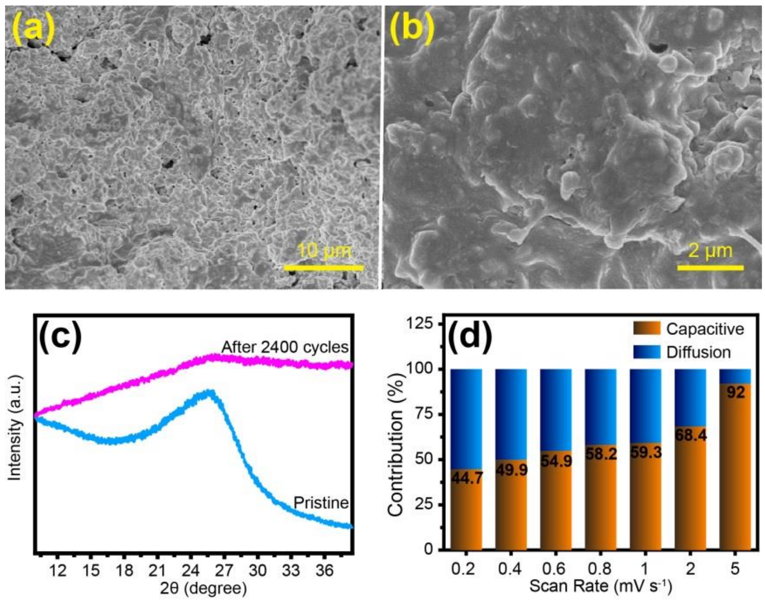

Figure. S10 (a), (b) SEM images of CBC@G after 2400 cycles at 5C, (c) XRD patterns of CBC@G before and after 2400 cycles at 5C, (d) normalized contribution ratio of capacitive behavior and battery-behavior capacities at different scan rates of CBC.

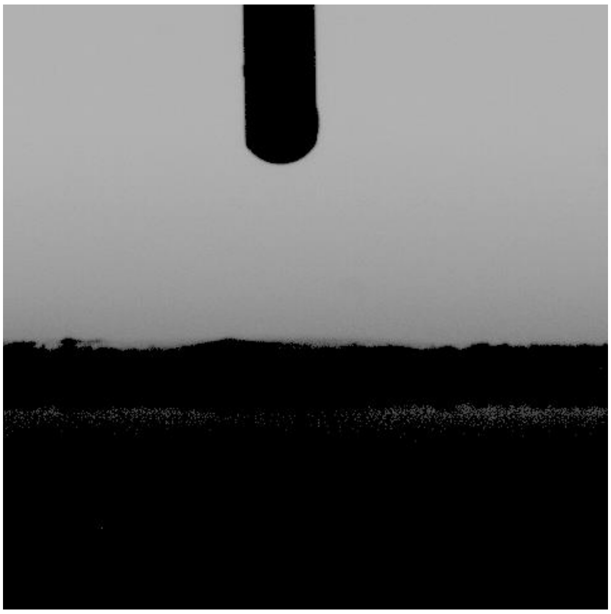

Figure. S11 Contact angle to 0.8 M KPF_6 (EC/EDC=1/1) of CBC@G.

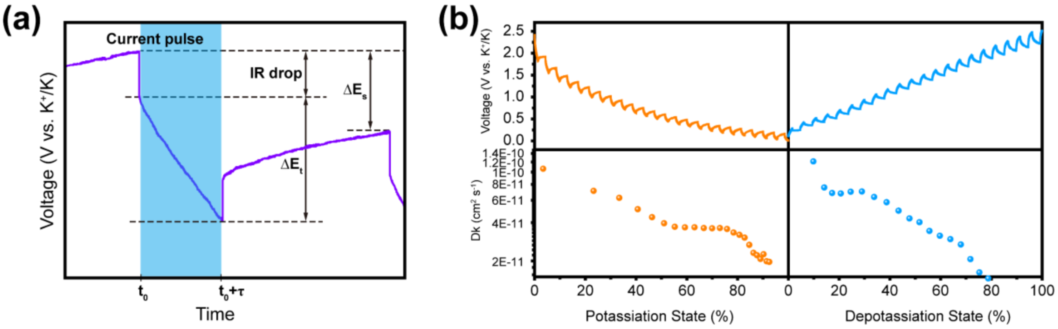

Figure. S12 (a) Schematic of the calculation of diffusion coefficient (D_k) using the GITT technique, (b) GITT curves and D_k of CBC@G.

$$D_K = \frac{4}{\pi\tau}\left(\frac{m_b V_M}{M_B S}\right)^2 \left(\frac{\Delta E_S}{\Delta E_t}\right)^2 \qquad \left(t \ll \frac{L^2}{D}\right)$$

where τ is the pulse time (s), m_b is the mass of the active materials, M_B is the molar mass of the active material (12 g mol^{-1}), V_M represents the molar volume of the active material, S is the geometric area of the electrode, and ΔE_s and ΔE_t are defined as shown in the Figure S12a.

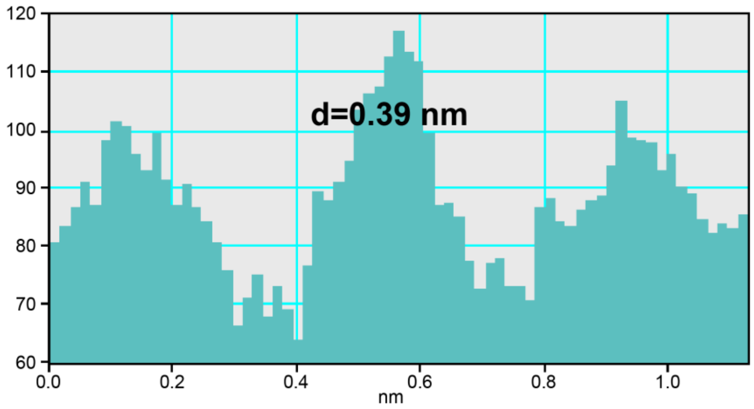

Figure. S13 The calculated interlayer spacing of **Figure 4f**.

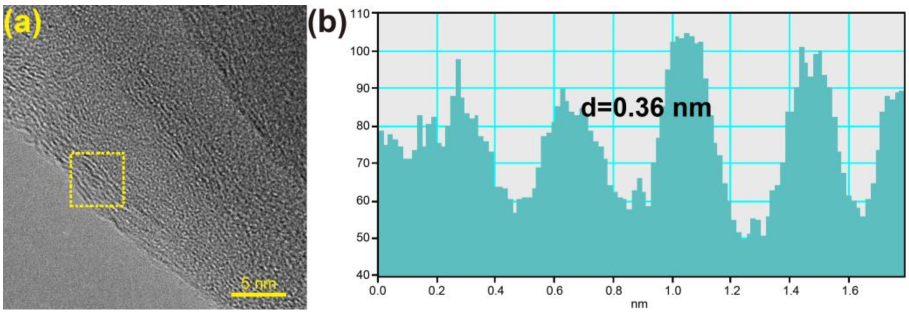

Figure. S14 (a) TEM image of fully charged CBC@G, (b) the calculated interlayer spacing.

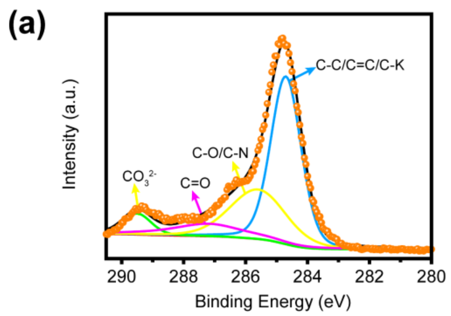

Figure. S15 (a) Deconvolution C 1s spectrum, (a) deconvolution N 1s spectrum of fully charged CBC@G.

As shown in Figure S15a, the C 1s spectrum contains four component peaks centered at 284.7, 285.6, 287.2, and 289.5 eV, corresponding to C−C/C=C/C-K, C−O/C−N, C=O, and CO_3^{2-}, respectively.[8] The presence of CO_3^{2-} peak may indicate that K_2CO_3 is one of the components of SEI. Further, the spectrum of O 1s (Figure S15b) can be deconvolved as C-O (530.7 eV), C=O (531.5 eV), K=O (531.7 eV) and CO_3^{2-} (533.1 eV), which confirms the analysis results of K 2p and C1s.

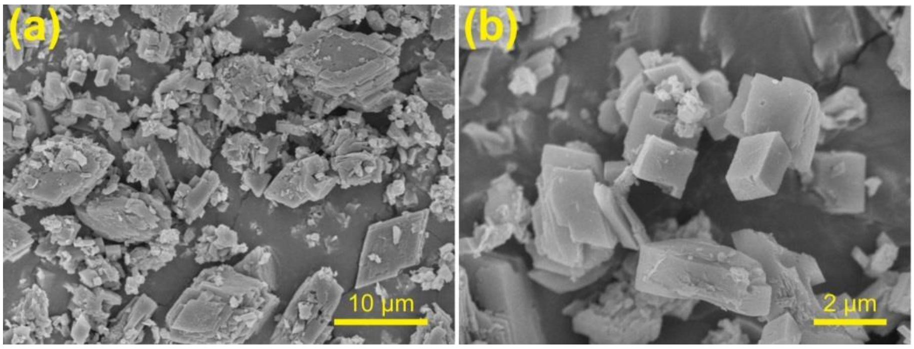

Figure. S16 SEM images of ACBC.

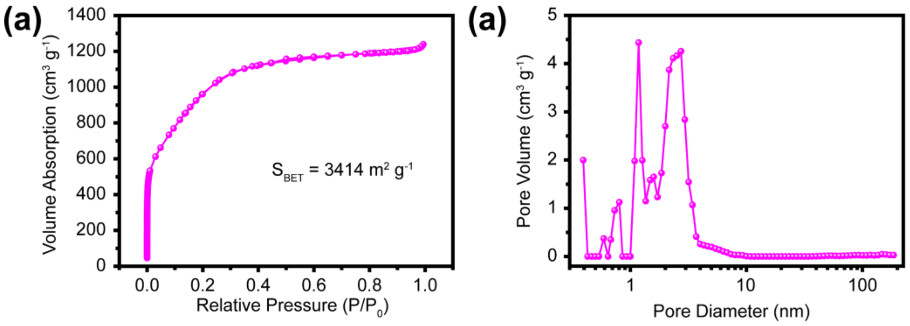

Figure. S17 (a) N_2 adsorption-desorption isotherm, (b) DFT pore size distribution of ACBC.

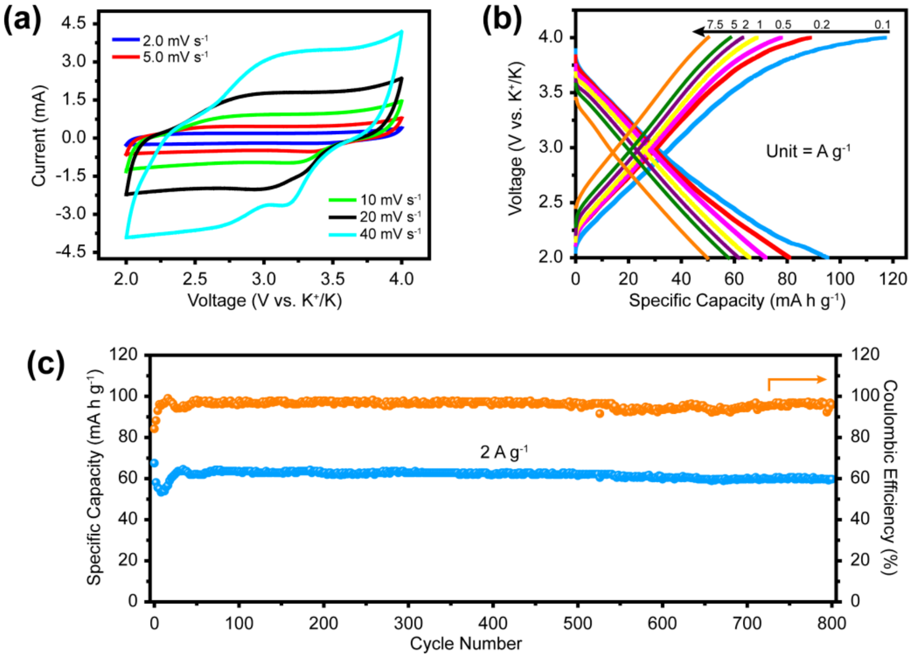

Figure. S18 Electrochemical performance of ACBC cathode. (a) CV curves at different scan rates, (b) GCD curves at different current densities and (c) cycling stability at 2 A g^{-1}.

As shown in Figure S16, KOH-activated CBC (ACBC) can still maintain the original nanoplate-stacked cubes morphology, but there are numerous debris spalling from the cube. Further, ACBC exhibits combined type I/IV isotherm with a significant H4 type hysteresis loop (Figure S17a), indicating the presence of abundant micropores and mesopores.[9] As a result, the BET specific surface area (S_{BET}) of ACBC is 3414 m^2 g^{-1}, with a more than 6.7 times increase in S_{BET} after KOH activation. Notably, the pore size distribution of ANHCS is concentrated at 1−4 nm (Figure S17b). Such a prominent pore size distribution is beneficial for ACBC cathode to adsorb more K^+.[10] To further investigate the electrochemical properties of ACBC-based cathode, a half-cell configuration with potassium wafer as the counter electrode is utilized. Figure. S18a exhibits the CV curves of ACBC at different scan rates in the potential range of 2.0–4.0 V. The approximate quasi-rectangular shape at different scan rates not only represents the dominant capacity of EDLC, but also indicates the excellent rate capability of ACBC. Notably, a pair of broad peaks in the CV curve at about 3–3.5 V is attributed to the additional pseudocapacitance contributed by heteroatoms (N and O). The GCD curves of ACBC at different current densities are shown in Figure S18b. The ACBC cathode delivers an ultrahigh reversible specific capacity of 95.2 mA h g^{-1} at 0.1 A g^{-1} and remains 49 mA h g^{-1} at an ultrahigh current density of 7.5 A g^{-1}. In addition, the ACBC cathode can maintain nearly 100% initial capacity with nearly 100% Coulombic efficiency after 800 cycles at a high current of 2 A g^{-1} (Figure S18c), exhibiting excellent cycling stability.

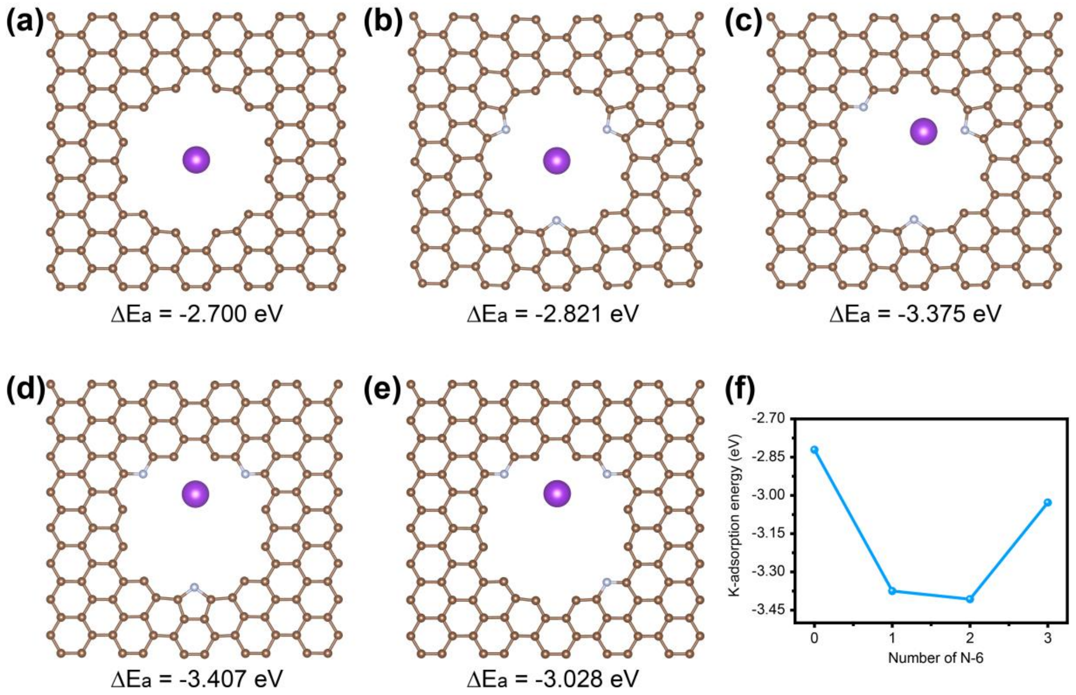

Figure. S19 Theoretical simulations of K-adsorption in different N-doped configurations. (a) defect, (b)3N-5, (c)1N-6/2N-5, (d) 2N-6/1N-5, (e) 3N-6; (f) K-adsorption energies of the graphene layers based on different N-doping modes.

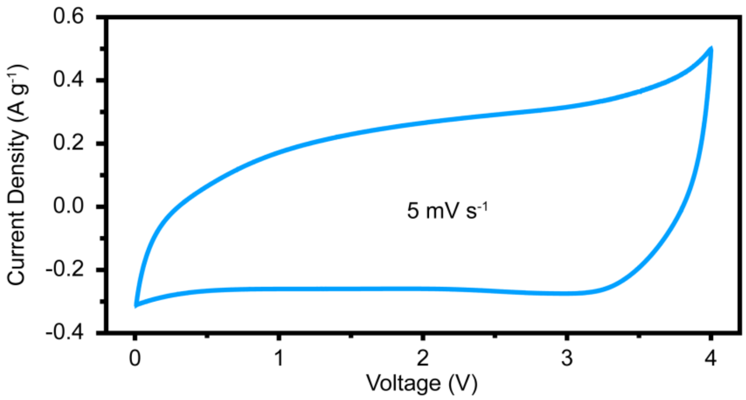

Figure. S20 CV curve of CBC@G//ACBC PIHCs at 5 mV s^{-1}.

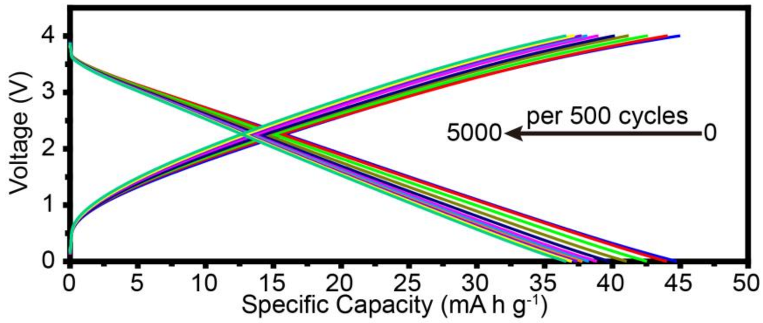

Figure. S21 GCD curves of CBC@G//ACBC PIHCs at different cycles (5 A g^{-1}).

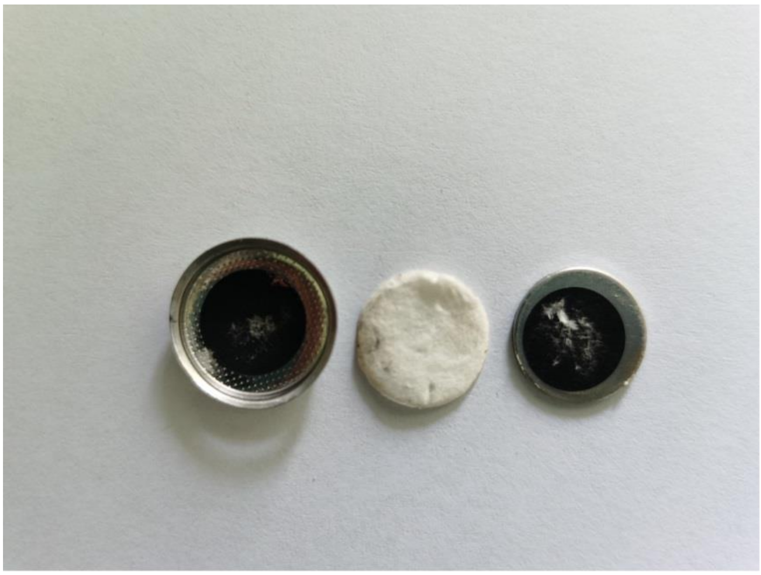

Figure. S22 Digital photograph of the disassembled parts of CBC@G//ACBC.

Table S1. Textual parameters of CBC and CBC@G.

Sample	$^{a}S_{BET}$	$^{b}V_{<2\ nm}$	$^{c}V_{<10\ nm}$	$^{d}V_{<50\ nm}$	$^{e}V_{>50\ nm}$	$^{f}V_{DFT}$
	($m^2\ g^{-1}$)	($cm^3\ g^{-1}$)				
CBC	509	0.076(14.4%)	0.404(76.4%)	0.032(6.0%)	0.017(3.2%)	0.529
CBC@G	524	0.075(9.9%)	0.337(44.3%)	0.172(22.6%)	0.176(23.2%)	0.76

a: The BET specific surface area; b: Pore volume of micropores calculated by DFT; c: Pore volume of 2 nm<*d*<10 nm calculated by DFT; d: Pore volume of 10 nm<*d*<50 nm calculated by DFT; e: Pore volume of macropores calculated by DFT; f: The total DFT pore volume.

Table S2. Surface element composition evaluated from XPS and relative contents of functional groups in N1s peaks of CBC and CBC@G.

Sample	C	O	N	N-6	N-5	N-Q
	(at.%)					
CBC	77.3	4.2	18.5	7.56	8.13	2.81
CBC@G	79.1	5.4	15.5	7.94	4.96	2.6

References

[1] Y. Liu, Y.-X. Lu, Y.-S. Xu, Q.-S. Meng, J.-C. Gao, Y.-G. Sun, Y.-S. Hu, B.-B. Chang, C.-T. Liu, A.-M. Cao, *Adv. Mater.* **2020**, *32*, 2000505.

[2] L. Fan, R. Ma, Q. Zhang, X. Jia, B. Lu, *Angew. Chem. Int. Ed.* **2019**, *58*, 10500-10505.

[3] N. Sun, Q. Zhu, B. Anasori, P. Zhang, H. Liu, Y. Gogotsi, B. Xu, *Adv. Funct. Mater.* **2019**, *29*, 1906282.

[4] X. Chen, H. Zhang, C. Ci, W. Sun, Y. Wang, *ACS Nano* **2019**, *13*, 3600-3607.

[5] J. Liu, T. Yin, B. Tian, B. Zhang, C. Qian, Z. Wang, L. Zhang, P. Liang, Z. Chen, J. Yan, X. Fan, J. Lin, X. Chen, Y. Huang, K. P. Loh, Z. X. Shen, *Adv. Energy Mater.* **2019**, *9*, 1900579.

[6] D. Qiu, A. Gao, Z. Xie, L. Zheng, C. Kang, Y. Li, N. Guo, M. Li, F. Wang, R. Yang, *ACS Appl. Mater. Interfaces* **2018**, *10*, 44483-44493.

[7] F. Pei, T. An, J. Zang, X. Zhao, X. Fang, M. Zheng, Q. Dong, N. Zheng, *Adv. Energy Mater.* **2016**, *6*, 1502539.

[8] Y. Lei, D. Han, J. Dong, L. Qin, X. Li, D. Zhai, B. Li, Y. Wu, F. Kang, *Energy Storage Mater.* **2020**, *24*, 319-328.

[9] T. J. Barton, L. M. Bull, W. G. Klemperer, D. A. Loy, B. McEnaney, M. Misono, P. A. Monson, G. Pez, G. W. Scherer, J. C. Vartuli, O. M. Yaghi, *Chem. Mater.* **1999**, *11*, 2633-2656.

[10] V. S. Kale, M. Hwang, H. Chang, J. Kang, S. I. Chae, Y. Jeon, J. Yang, J. Kim, Y.-J. Ko, Y. Piao, T. Hyeon, *Adv. Funct. Mater.* **2018**, *28*, 1803786.
